# Supplementary material for: A fractal kinetics SI model can explain the dynamics of COVID-19 epidemics
Source: PLoS One. 2020 Aug 11;15(8):e0237304. doi: 10.1371/journal.pone.0237304 (PMC7418974; doi:10.1371/journal.pone.0237304)
Supplement: S3 File — The fitting of the model 2 to the data in file was performed using the python lmfit module [23]. For each country the starting values for the parameter were a = [60, 600, 6000], c = [10, 80, 800], h = [2.5, 4.0] i.e 18 possible triplets. For each one of these 18 starting “points” we applied a basin-hopping algorithm with a Nelder-Mead local optimizer and independently a Levenberg-Marquardt minimization algorithm. (Basin-hopping is a two-phase method that combines a global stepping algorithm with local minimization at each step.) Thus, for each country we obtained 36 estimations for the values of the parameters a, c, h and among them we choose the one with the minimum chi-squared χ2. Here we include the fit statistics for the countries appearing in Fig 1. (DOCX) [file pone.0237304.s003.docx]

**China**

[[Model]]

Model(fkin)

[[Fit Statistics]]

# fitting method = Nelder-Mead

# function evals = 1255

# data points = 73

# variables = 3

chi-square = 4.8280e-10

reduced chi-square = 6.8971e-12

Akaike info crit = -1873.15744

Bayesian info crit = -1866.28606

[[Variables]]

a: 2012.97109 +/- 825.051570 (40.99%) (init = 600)

c: 16302.2827 +/- 179.437218 (1.10%) (init = 800)

h: 3.48943013 +/- 0.12861623 (3.69%) (init = 2.5)

[[Correlations]] (unreported correlations are < 0.100)

C(a, h) = 0.995

C(c, h) = 0.727

C(a, c) = 0.686

###########

**Korea, South**

[[Fit Statistics]]

# fitting method = Nelder-Mead

# function evals = 2044

# data points = 73

# variables = 3

chi-square = 1.2666e-09

reduced chi-square = 1.8094e-11

Akaike info crit = -1802.75015

Bayesian info crit = -1795.87877

[[Variables]]

a: 2.7328e+10 +/- 440.955752 (0.00%) (init = 600)

c: 5268.94366 +/- 29.1350130 (0.55%) (init = 800)

h: 7.08193252 +/- 0.00552258 (0.08%) (init = 2.5)

[[Correlations]] (unreported correlations are < 0.100)

C(c, h) = 0.593

###########

**United States**

[[Fit Statistics]]

# fitting method = Nelder-Mead

# function evals = 8259

# data points = 73

# variables = 3

chi-square = 3.0197e-10

reduced chi-square = 4.3138e-12

Akaike info crit = -1907.41471

Bayesian info crit = -1900.54333

[[Variables]]

a: 21317410.6 +/- 132.381036 (0.00%) (init = 600)

c: 108.041344 +/- 0.79852436 (0.74%) (init = 80)

h: 4.45145993 +/- 6.0935e-04 (0.01%) (init = 2.5)

[[Correlations]] (unreported correlations are < 0.100)

C(c, h) = 0.989

###########

**Italy**

[[Fit Statistics]]

# fitting method = Nelder-Mead

# function evals = 3059

# data points = 64

# variables = 3

chi-square = 2.0977e-08

reduced chi-square = 3.4389e-10

Akaike info crit = -1391.67729

Bayesian info crit = -1385.20064

[[Variables]]

a: 273552.071 +/- 395.936537 (0.14%) (init = 60)

c: 200.606786 +/- 2.29431511 (1.14%) (init = 10)

h: 3.79619102 +/- 0.00214082 (0.06%) (init = 2.5)

[[Correlations]] (unreported correlations are < 0.100)

C(c, h) = 0.953

C(a, h) = 0.173

###########

**Spain**

[[Fit Statistics]]

# fitting method = Nelder-Mead

# function evals = 3162

# data points = 63

# variables = 3

chi-square = 2.9214e-08

reduced chi-square = 4.8691e-10

Akaike info crit = -1347.97961

Bayesian info crit = -1341.55021

[[Variables]]

a: 2174222.82 +/- 267.635504 (0.01%) (init = 6000)

c: 99.8577483 +/- 1.55371914 (1.56%) (init = 80)

h: 4.18076247 +/- 0.00212787 (0.05%) (init = 2.5)

[[Correlations]] (unreported correlations are < 0.100)

C(c, h) = 0.977

###########

**France**

[[Fit Statistics]]

# fitting method = leastsq

# function evals = 779

# data points = 71

# variables = 3

chi-square = 4.3850e-08

reduced chi-square = 6.4485e-10

Akaike info crit = -1499.56712

Bayesian info crit = -1492.77908

## Warning: uncertainties could not be estimated:

c: at boundary

[[Variables]]

a: 27.2872296 (init = 60)

c: 5.5056e-09 (init = 80)

h: 1.29788920 (init = 2.5)

###########

**Germany**

[[Fit Statistics]]

# fitting method = Nelder-Mead

# function evals = 3254

# data points = 68

# variables = 3

chi-square = 3.6584e-09

reduced chi-square = 5.6283e-11

Akaike info crit = -1601.91041

Bayesian info crit = -1595.25189

[[Variables]]

a: 5081709.28 +/- 316.294727 (0.01%) (init = 6000)

c: 233.413859 +/- 2.84598325 (1.22%) (init = 800)

h: 4.31338939 +/- 0.00163292 (0.04%) (init = 2.5)

[[Correlations]] (unreported correlations are < 0.100)

C(c, h) = 0.977

###########

**Australia**

[[Fit Statistics]]

# fitting method = Nelder-Mead

# function evals = 5874

# data points = 69

# variables = 3

chi-square = 5.4973e-10

reduced chi-square = 8.3292e-12

Akaike info crit = -1757.34383

Bayesian info crit = -1750.64151

[[Variables]]

a: 5.8723e+11 +/- 497.858390 (0.00%) (init = 6000)

c: 2629.99808 +/- 34.4559360 (1.31%) (init = 800)

h: 7.12806858 +/- 0.00346841 (0.05%) (init = 2.5)

[[Correlations]] (unreported correlations are < 0.100)

C(c, h) = 0.926

###########

**Malaysia**

[[Fit Statistics]]

# fitting method = Nelder-Mead

# function evals = 3951

# data points = 70

# variables = 3

chi-square = 6.8905e-11

reduced chi-square = 1.0284e-12

Akaike info crit = -1929.27559

Bayesian info crit = -1922.53011

[[Variables]]

a: 20774718.0 +/- 426.482577 (0.00%) (init = 6000)

c: 3954.67789 +/- 47.0892775 (1.19%) (init = 80)

h: 4.69917585 +/- 0.00222703 (0.05%) (init = 4)

[[Correlations]] (unreported correlations are < 0.100)

C(c, h) = 0.960

###########
