# Supplementary material for: A fractal kinetics SI model can explain the dynamics of COVID-19 epidemics
Source: PLoS One. 2020 Aug 11;15(8):e0237304. doi: 10.1371/journal.pone.0237304 (PMC7418974; doi:10.1371/journal.pone.0237304)
Supplement: S1 Appendix — (PDF) [file pone.0237304.s006.pdf]

S1 Appendix. Structural Identifiability of the Fractal Kinetic SI Model. The subject of identifiability of an ODE Model has attracted some attention in the Biomodeling community recently [8, 24–26]. Non-Identifiability typically poses problems for large scale models comprising several ODEs with a lot of parameters, but also small scale models is possible to "suffer" from it. Following the definition of [8]" Structurally identifiable addresses the question of whether quantification of parameters is possible from a given set of ideal, noise-free input-output data. A priori Structural Identifiability

is thus a necessary condition for quantifying any model parameter p from real data". Below we present a Structural Identifiability analysis using the Taylor series method and show that the Fractal kinetic SI Model 1 is Structuraly Identifiable. Fractal Kinetic models typically have an initial condition of the form $y(t=1)=A$ where A is known. Initially, we transform Eq. 1 using the independent variable change $t=\tau+1$. Thus, the model is written in the convenient form

$$\frac{dI}{d\tau}=\frac{a}{(\tau+1)^h}I(1-I)-bI \tag{6}$$

with initial condition $I(0)=\xi_0$

We assume that the observation function $I(\tau;a,b,h)$ is analytic in a neighborhood of $\tau=0$, i.e. I is infinitely differentiable with respect to τ on this neighborhood. Under this assumption,$I(\tau;a,b,h)$ and its successive time derivatives can be expanded in a Taylor series around $\tau=0$ and evaluated in terms of the model parameters. Since the coefficients in the Taylor series expansion are unique, the problem of Structural Identifiability reduces to determining if the set of nonlinear algebraic equations resulting from the Taylor expansion is solvable for the parameters a,b,h. Note that since the function $I(\tau)$ is the quantity that we observe it is assumed as known and, thus, its Taylor expansion coefficients are also assumed to be known. The Taylor formula for $I(\tau)$ is:

$$I(\tau)=I(0)+\frac{dI}{d\tau}(0)+\frac{1}{2}\frac{d^2I}{d\tau^2}(0)+\cdots \tag{7}$$

and using the initial condition $I(0)=\xi_0$ and the Eq 6 we conclude that

$$\frac{dI}{d\tau}(0)=-a\xi_0^2+a\xi_0-b\xi_0 \tag{8}$$

Since the time derivatives of $I(\tau)$ are assumed known we will use the notation $\frac{dI}{d\tau}(0)=\xi_1$, $\frac{d^2I}{d\tau^2}(0)=\xi_2$ etc. Thus, successive differentiation of Eq 6 will lead to the following nonlinear algebraic system

$$\xi_1=-a\xi_0^2+a\xi_0-b\xi_0 \tag{9}$$

$$\xi_2=ah\xi_0^2-ah\xi_0-2a\xi_0\xi_1+a\xi_1-b\xi_1 \tag{10}$$

$$\xi_3=-ah^2\xi_0^2+ah^2\xi_0-ah\xi_0^2+4ah\xi_0\xi_1+ah\xi_0-2ah\xi_1-2a\xi_0\xi_2-2a\xi_1^2+a\xi_2-b\xi_2 \tag{11}$$

Using a Computer Algebra system (for example Mathematica [27]) we solve the above equations for a,b,h. We find two solutions out of which one is rejected due to the constrain that a,b,h should be non negative. We have also investigated the above system numerically and for a large range of ξ_i values and never found more than one triplet that solves the system and simultaneously satisfies the non negativity restriction. Thus, we conclude that the Fractal Kinetic SI model is structuraly identifiable.
