# Supplementary figures and images for: A fractal kinetics SI model can explain the dynamics of COVID-19 epidemics
Source: PLoS One. 2020 Aug 11;15(8):e0237304. doi: 10.1371/journal.pone.0237304 (PMC7418974; doi:10.1371/journal.pone.0237304)

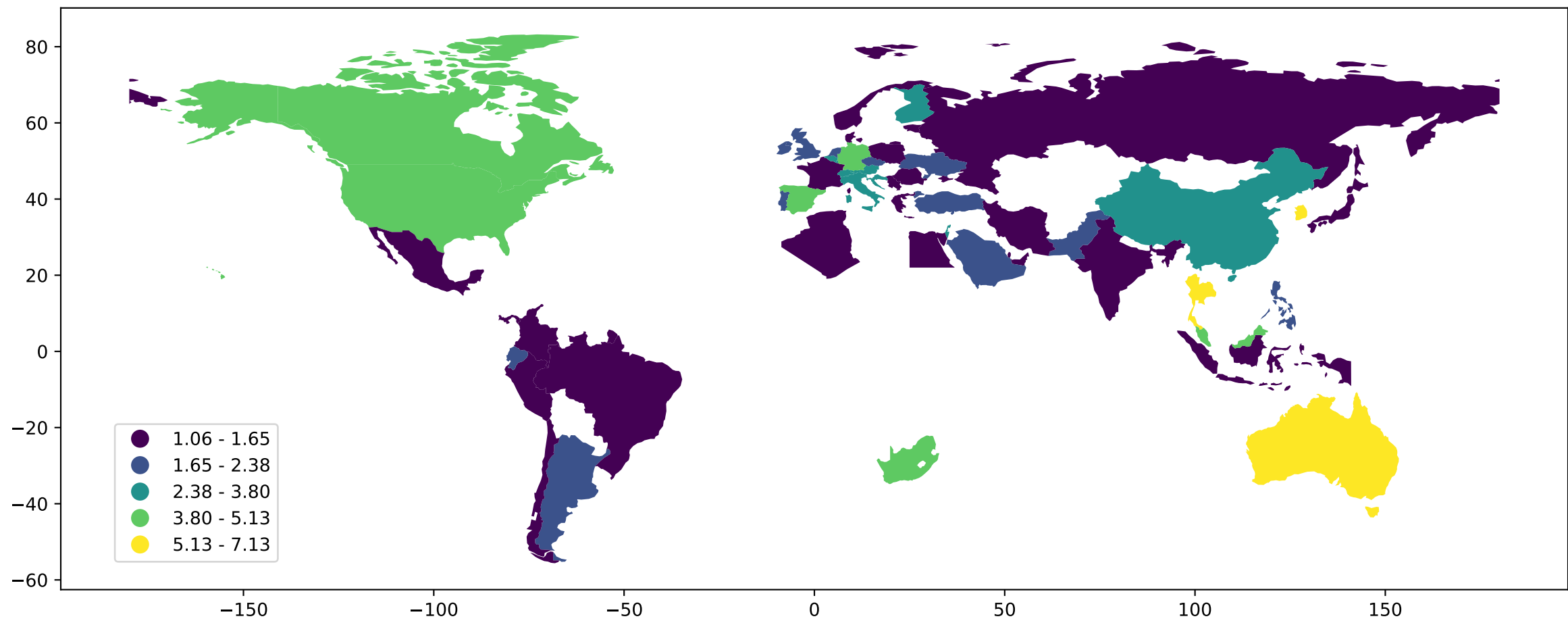

Supplement: S1 Raw images — (PDF) [file pone.0237304.s007.pdf]
