# Supplementary material for: A fractal kinetics SI model can explain the dynamics of COVID-19 epidemics
Source: PLoS One. 2020 Aug 11;15(8):e0237304. doi: 10.1371/journal.pone.0237304 (PMC7418974; doi:10.1371/journal.pone.0237304)
Supplement: Attachment — Submitted filename: Response_To_Reviewers.docx [file pone.0237304.s008.docx]

**RESPONSE TO REVIEWERS**

*Reviewer #1*

*In this paper, the authors adopt a quite simple fractal SI model to the cumulative data of several countries facing with COVID-19 epidemics. Basically, this is a worthwhile try, but more detailed*

*analyses have to be carried out before acception.*

We thank the Reviewer for his assessment as well as for his time and effort.

*(1) The fractal SI model is not fully justified. Though there is a breif mention that fractal kineitcs "describe reaction dynamics when imperfect mixing and segregation of the reactants". But more detailed connections with epidemics need to be clarfied.*

We have considerably extended the discussion and added two full paragraphs (page 2 lines 36 -55 of the revised manuscript) and several clarifying sentences (page 2 lines 63-66, page 3 ) where we explain the motivation behind the fractal kinetics SI model. *(2) Statistical analysis on the model paramters and their influence on the prediction are missing.*

We have included in supplementary materials File S3 and S4 with all the details of the fitting of our model parameters and indicators for the goodness of fit such as the chi-squared values and the Akaike information criterion for model selection.  *(3) The confidence region for cumulative data is quite narrow. Daily data is more sensitive.*

Cumulative data smooth out noise and fluctuation due to imprecise measurements. This is the reason that we prefer them for the estimation of the model parameters

*(4) Would the replacement of differential equation in (2) by difference equation resolve the diveregence problem, when t goes to zero?*

A discrete model based on difference equations is indeed a viable alternative. In Fractal kinetics ODE modeling the problem is circumvented by the standard choice of initial conditions at t=1 i.e. y(t=1)=y0=known. And of course, since time is a continuous variable an ODE model is a little bit more natural.  *(5) All data used in the manuscript should be made publically available, either in SI or on a webserver.*

All data files are included in the supplementary materials and information on how to access them from the original sources is included in the manuscript (page 5 line 66-72) *(6) PCA is an interesting study. Though the origin and consequence of three clusters appearing in Fig. 3 requires more explanations.|*

We also feel that PCA is an interesting way to identify common factors in this case. Although the clusters are obvious and that is why we report them only speculations can be made about their origin. We believe that it is affected by the phase of the epidemic each country is (e.g if it is near the plateau or not) and, also, on the degree of self-organization of the society in response to the epidemic. But at this point this is just an assumption.

*(7) Does h have some correlation with the basic reproduction number? What's the physical meaning of a ranking based on h in Fig. 2?*

These are rather different quantities. Τhe basic reproduction of an infection can be thought of

as the expected number of cases directly generated by one case in a population where all individuals are susceptible to infection. The definition describes the state where no other individuals are infected or

immunized and some definitions, such as that of the Australian Department of Health, add absence of "any deliberate intervention in disease transmission".

The h exponent, on the other hand, aims to quantify exactly this deliberate intervention and the fact that not all individuals, as time progresses, are susceptible to infection. Finally, knowledge of h does not suffice to determine the basic reproduction number (which is a quantity that depends also on the rate b to say the least).

The ranking based on h is motivated by the fact that the ``fractal'' exponent h quantifies the self-organization of the societies against the pandemic through social distancing, lockdowns and flight restrictions. Thus, high values of the h exponent is an indication for the successful application of preventive measures for a country. We have included that info in lines 60-75 and 108-111 of the revised manuscript.

*(8) A full list on the countries studied in the manuscript is needed. Otherwise, Fig. 2 and 3 become meaningless.*

This list is included in Supplementary materials S5 along with yhe Principle components for eavh country.

*Reviewer #2: The idea and topic is interesting.*

We thank the Reviewer for his assessment.

*However, there are many things missing and some caveats. The authors argue that the model fits very well to the data. However, there are many models that can fit very well to the same datasets. Somebody can argue that the best one is the one with the smallest SSR.*

The SSR neglects the fact that the number of adjustable parameters may differ between two models and thus it is not a good quanity for modelk comparison. The Akaike information Criterion is more suitable. In any case, in Supplementary materials S4 we include Fitting results including estimated parameter values, chi-squared $\chi^2$, reduced chi-squared and Akaike information Criterion for the 55 Countries that we have studied.

*In addition, it is not clear if the parameter values are unique. The authors need do an identifiability analysis.*

In Supplementary materials S! Appendix we show that the Fractal SI model is structurally identifiable.

*The authors need to explain better the justification for the parameter h.*

We have considerably extended the discussion and added two full paragraphs (page 2 lines 36 -55 of the revised manuscript) and several clarifying sentences (page 2 lines 63-66, page 3 ) where we explain the motivation behind the fractal kinetics SI model.
