# Supplementary material for: A fractal kinetics SI model can explain the dynamics of COVID-19 epidemics
Source: PLoS One. 2020 Aug 11;15(8):e0237304. doi: 10.1371/journal.pone.0237304 (PMC7418974; doi:10.1371/journal.pone.0237304)
Supplement: Attachment — Submitted filename: Response_To_Reviewers.docx [file pone.0237304.s009.docx]

**RESPONSE TO REVIEWERS**

*Reviewer #1: I think the authors have properly addressed all of my previous concerns in the revision. So I recommend its publication in PLoS One.*

We thank the Reviewer for his assessment as well as for his time and effort.

*Reviewer #2: The authors improved the paper*.

We thank the Reviewer for his assessment

*The PCA is not clear. Please add additional comments to understand the aims of using it.*

We have included one full paragraph (lines 144-162 of the revised manuscript) and 3 references with our motivation and several details of the PCA analysis. As this is a novel application of the technique, we hope that at least our motivation is clear.

*The fitting process in appendix S3 is local. What about the global minimum?*

Actually, the fitting process in appendix S3 is not local and we have included a line in the manuscript to clarify that. We have used 18 different triplets as starting points. For each one of these 18 starting points" we applied a basin-hopping algorithm and independently a

Levenberg-Marquardt minimization algorithm. Basin-hopping is a two-phase method

that combines a global stepping algorithm with local minimization at each step. It is considered a global optimization method (see for example <https://docs.scipy.org/doc/scipy/reference/optimize.html>). In our case a Nelder-Mead optimizer for the local step.

*Sturctural identifiability is only a necessary condition for “practical” identifiability; it is not sufficient. Since one of the main points of the article is the h value and the fitting process, it is important to show the practical identifiability of the 3 parameters with the available data.*

We have included a Structural identifiability analysis in the revision of the manuscript following the reviewer’s suggestion as we found that the concept is theoretically interesting. Indeed, Sturctural identifiability is a necessary condition for “practical” identifiability but not sufficient. Practical identifiability may be a problem when parameters are estimated from the numerical solution of ODEs.

Note, however, that in our case we are fortunate enough to obtain an exact solution of the ODE model ,i.e. Eq 4. And we estimate the parameters including the h value by fitting the data to this algebraic equation. Thus, the problem of practical identifiability, i.e. the question of whether the model parameters can be estimated from the data becomes in this case equivalent to the goodness of fit problem discussed in the previous question.
